# An investigation of the change in alignment between the Health Star Rating Scheme and the Nova Food Processing Classification System and the Australian Dietary Guidelines

**DOI:** 10.1017/S1368980026102419

**Published:** 2026-03-27

**Authors:** Sandra Brastein, Julie Woods, Sarah Dickie, Mark Andrew Lawrence

**Affiliations:** 1 Institute for Physical Activity and Nutrition (IPAN), Deakin University, Burwood, Australia; 2 Monash University, Notting Hill, Australia

**Keywords:** Front-of-package labelling, Health Star Rating, Ultra-processed foods, Nova, Australian Dietary Guidelines

## Abstract

**Objective:**

Front-of-package labelling informs consumers about the ‘healthiness’ of foods based on different classification schemes. These schemes reflect competing worldviews for assessing a food’s healthiness, represented by nutrient-, food- or diet-based indices. The Health Star Rating scheme (HSR) has been criticised for failing to appropriately score unhealthy products. Updates to the HSR algorithm were implemented over a two-year period from November 2020. This study investigated alignment between a nutrient-based scheme (HSR), food-based scheme (Nova food processing system) and diet-based scheme (Australian Dietary Guidelines (ADG)).

**Setting:**

Mintel Global New Products Database

**Participants:**

Retail foods displaying HSR launched or updated onto the Australian market between November 2020 and June 2023.

**Design:**

Products were categorised according to the ADG and Nova, descriptive statistics performed for each category and proportion displaying HSR ≤ 2·0 and ≥ 2·5 calculated for discretionary foods, five food group foods, ultra-processed foods (UPF) and non-UPF. Agreement between categories obtained by Kappa.

**Results:**

Median HSR for UPF and discretionary foods were 3·5 and 2·5, respectively, and 73·7 % of UPF and 58·2 % of discretionary foods displayed HSR ≥ 2·5. Agreement between HSR and Nova was none to slight (k = 0·09, P < 0·001) and HSR and ADG was fair (k = 0·38, P < 0·001). Between 2020–2023, the proportion of UPF displaying HSR ≥ 2·5 increased from 60·2 % to 78·5 % and for discretionary foods 47·0 % to 62·5 %.

**Conclusion:**

The HSR algorithm calculates ‘healthy’ HSR (≥ 2·5) for a high proportion of UPF and discretionary foods. The HSR’s nutrient-based approach to translate food-and diet-based nutrition recommendations into accurate food ‘healthiness’ assessments is still problematic.

Dietary risk factors are among the leading contributors to the global burden of disease^(1)^. Many countries have developed dietary guidelines that recommend dietary choices to help prevent malnutrition^(2)^. The WHO has recommended policy interventions to promote healthy diets and reduce the prevalence of diet-related non-communicable diseases, including marketing restrictions on unhealthy foods; food reformulation to reduce ‘risk’ nutrient composition; food taxes and subsidies; regulation of health claims; and implementation of front-of-package labelling (FOPL)^(3–5)^. FOPL can guide consumers towards healthier food choices by displaying relevant nutrition information in an easily understood format^(6)^. The design of FOPL requires a metric to assess a food’s ‘healthiness’; however, there are competing worldviews towards the most appropriate assessment metric^(7,8)^. Nutrition classification schemes can draw on three main nutrition exposure indices to inform the metrics used to assess a food’s healthiness:

- *nutrient-based indices* use an algorithm based on a nutrient profiling model to assess a food’s healthiness by calculating its content of ‘risk’ nutrients and ‘beneficial’ components^(7,9)^. The Health Star Rating (HSR) is a voluntary FOPL scheme in Australia and New Zealand which assigns or deducts points according to a food’s content of energy, saturated fat, sodium and total sugars; and fruit, vegetables, nuts, legumes, fibre and protein; with final ratings between 0·5 (least healthy) and 5 stars (most healthy)^(10,11)^;
- *food-based indices* consider the chemical composition and physical structure of foods by assessing the degree and purpose of processing. The Nova classification system is the most widely used in research and policy^(12,13)^. Nova classifies foods into one of four categories: unprocessed or minimally processed foods (MPF), processed culinary ingredients (PCI), processed foods (PF) and ultra-processed foods (UPF)^(14)^. Consumption of UPF is associated with many adverse health outcomes^(15–17)^;
- *diet-based indices* assess healthiness in context of a food’s place within a dietary pattern by categorising foods into either recommended foods or foods for which consumption should be limited. Food-Based Dietary Guidelines such as the Australian Dietary Guidelines (ADG) provide guidance on amounts, types and variety of foods and food groups that are associated with good health and reduced risk of all forms of malnutrition^(18)^.

Misalignments have been reported between nutrient-, food- and diet-based indices in informing the assessment of foods’ healthiness^(7)^. Most studies evaluating the HSR against Nova have reported discrepancies, with 55·0–76·9 % of UPF products receiving HSR ≥ 2·5^(7,19,20)^ or ≥ 3·5^(21,22)^, effectively classifying these foods as healthy. These inconsistencies have been attributed to the discord between the reductionist nature of nutrient-centric FOPL schemes and the holistic food-based paradigm underpinning Nova^(7,8)^ and demonstrate the issues that arise because the HSR scheme does not consider processing when assessing healthiness of foods.

Studies evaluating the HSR against the ADG report that 17·4–56·7 % of all discretionary foods received ‘healthy’ HSR of ≥ 2·5^(7,8,19)^ or ≥ 3·5^(23)^. The inconsistency of these results is likely due to the lack of an official ‘healthy’ cut-off score, resulting in researchers adopting a variety of arbitrary cut-offs to differentiate between ‘healthy’ or ‘unhealthy’ foods. HSR were also overrepresented on unhealthy foods, with UPF or discretionary foods significantly more likely to display a HSR compared to MPF or five food group (FFG) products (*P* < 0·001)^(19,20)^; and both discretionary and FFG products had a range of 0·5–5 stars, which suggests the HSR may incorrectly score both healthy and unhealthy products^(8)^. Not only do studies report discrepancies in alignment between the HSR and the ADG or Nova, but parents have also reported they find the HSR unhelpful and confusing when selecting foods for their children^(24)^.

As a result of criticism regarding its calculation of number of stars able to be displayed on certain foods^(7)^, updates to the HSR algorithm were implemented over a two-year period from November 2020, with the aim to bring the scheme more in line with dietary guidelines^(11)^. The changes were mainly technical adjustments to the nutrient criteria, as well as removing the option for an energy-only icon and awarding automatic HSR for specific products, such as an automatic 5-star rating for minimally processed fruit and vegetables and plain water, and 4·5 stars for unsweetened flavoured waters^(11)^. The original HSR algorithm has previously been evaluated against both Nova and the ADG^(7)^; however, the updated algorithm has not been comprehensively evaluated to determine whether the changes have reduced the inconsistencies between the HSR, Nova and the ADG when assessing a food’s healthiness. The introduction of new products into the food system also requires further research to evaluate the updated algorithm based upon the current food market and as a continuation of performance monitoring of the HSR scheme.

This study aimed to investigate changes in the alignment of food healthiness ratings between the HSR scheme, the Nova food processing classification system and the ADG during the period of implementation following adjustments to the HSR scheme’s algorithm. The HSR algorithm was expected to improve alignment for some food product categories, but the underpinning reductionist approach was predicted to continue to produce inconsistencies with the holistic approach that informs food- and diet-based schemes in their food healthiness ratings.

## Methods

### Study design and data collection

Food and beverage product data were collected from the Mintel Global New Products Database (Mintel), an online industry database containing data on new and updated packaged food and beverage products released onto the global market^(25)^. Product data are collected by ‘field associates’ (shoppers) who monitor market activity across key retail distribution channels and ship products biweekly to Mintel, enabling product data to be entered into the database within approximately one month of market launch, or in some cases, even prior to the official launch date^(24)^. Mintel is comprehensive, with an average of 8297 new and updated food and drink products entered in the database per year between December 2020 and November 2025^(26)^. The accuracy of data in Mintel is, however, reliant upon input of correct HSR and product data by shoppers. It also does not represent the total available food supply, as Mintel only collects information on products flagged as new or updated. A text search for ‘Health Star Rating’ was performed, with criteria limited to food and beverage products on the Australian market launched or updated between 1 November 2020 and 30 June 2023. This timeframe was chosen to capture products displaying HSR that were released or updated from the initial implementation date of the updated HSR algorithm (15 November 2020) and following the final date for implementation (15 November 2022). Mintel categories of ‘Baby formula’, ‘Growing up milks’, ‘Alcoholic beverages’, as well as any formulated sports foods and foods for special medical purposes found in the dataset were excluded as these are ineligible to display a HSR^(27)^. Product data including name, ingredients, nutritional composition, Mintel food category and HSR were extracted for coding and analysis. Products comprising multiple items with differing HSR (such as variety packs) were omitted from analyses. Missing ingredient information was obtained from manufacturer or supermarket websites.

### Data analysis

Mintel food categories were used to initially categorise products. Extracted product data were then categorised according to both the ADG and the Nova classification system using procedures documented and described in previous research^(7,28)^.

#### Australian Dietary Guidelines

Products were classified according to the ADG into one of ten categories: Grain foods; Fruit; Vegetables; Meat/eggs/tofu/nuts/seeds/legumes; Milk/yoghurt/cheese/alternatives; Mixed meals or food mixes; Discretionary foods; Culinary ingredients; Formulated supplementary foods; and Water. Discretionary foods were identified using a list of foods developed by the Australian Bureau of Statistics for the 2011–2013 Australian Health Survey^(7,29)^. Using a binary system, foods were further classified as either nutritious FFG foods in line with dietary guidelines or non-nutritious discretionary foods^(7,30)^.

#### Nova food processing classification system

Product ingredient lists from Mintel were used to classify products into one of the four Nova food processing classification system categories, according to degree and purpose of processing: MPF, PCI, PF and UPF^(14)^. UPF were identified by the presence of at least one industrial food substance or cosmetic additive (markers of ultra-processing, MUP) from a list used in previous research^(28)^.

An inter-rater cross-check of 20 % of the sample was conducted by three team members (ML, JW and SD) to assess the reliability of the allocated food processing and ADG group classifications. Where there were uncertainties in classifications among team members, a conservative approach was adopted, and products were classified as non-UPF or FFG in such instances.

### Statistical analysis

Statistical analyses were conducted using IBM SPSS Statistics Version 29.0 (SPSS Inc.), with *P*-value set to 0·05 for all analyses. Descriptive statistics were produced for the number and proportion of products in each ADG and Nova category, and HSR frequency, mode, median and interquartile range for the total sample and for each ADG and Nova category. Categories were also combined to compare discretionary foods and FFG foods, and UPF and non-UPF (comprising MPF, PCI and PF). Number and proportion of products displaying HSR below 2·5 stars or above or equal to a cut-off of 2·5 stars were calculated for discretionary and FFG foods, and for UPF and non-UPF. This was a logic-informed cut-off based upon previous research, considered as a ‘healthy pass’, as this corresponds to a scholastic pass mark of 50 %^(7,19)^. For optimal alignment, FFG foods and non-UPF would be expected to display HSR ≥ 2·5 stars, while discretionary foods and UPF would be expected to display HSR < 2·5 stars^(7)^.

A Kruskal–Wallis test was performed to compare median HSR and to identify significant differences between years and trends over the implementation period for the updated algorithm. HSR frequency, mode, median and interquartile range were also calculated for each year of the total sample, each ADG and Nova category, and for discretionary and FFG foods, UPF and non-UPF. As data were not normally distributed, Mann–Whitney U tests were used to determine any significant differences between median HSR for discretionary and FFG foods, and for UPF and non-UPF foods. Agreement between the HSR and Nova, and the HSR and the ADG was determined using Cohen’s Kappa coefficient and interpreted according to Landis and Koch: < 0·00 poor; 0·00–0·20 slight; 0·21–0·40 fair; 0·41–0·60 moderate; 0·61–0·80 substantial; 0·81–1·00 almost perfect^(31)^.

### Sub-analyses

Sub-analyses of the complete dataset were conducted for the Mintel categories ‘Juice drinks’ and ‘Fruit and vegetables’ to investigate effects of the lengthy two-year implementation period on HSR for these products. Median HSR and number and proportion of ‘Juice drinks’ scoring ≤ 3·5 and > 3·5 stars for each year were calculated, and a sub-analysis was performed by adjusting all products in this category with HSR of 5·0 stars down to 3·5 stars, in line with the maximum attainable HSR for juice products under the updated algorithm (except for tomato juice, which can receive 4·0 stars). These results were compared with median HSR by year for ‘Fruit and vegetables’ to assess differences in uptake of the new algorithm according to potential benefit to the product category, as fruit and vegetable products without added sugar or salt automatically receive a 5-star rating under the updated criteria.

Detailed product data were extracted for food categories that are normally major sources of sugars in cases where these had HSR ≥ 2·5, but were also classified as discretionary foods and/or UPF. This was to investigate the reason for these products scoring higher HSR despite the stricter sugar criteria under the updated HSR algorithm. Product categories included in these analyses were ‘Carbonated soft drinks’, ‘Sweet spreads’ and ‘Sweeteners and sugar’. A similar analysis was conducted for ‘Nutritional drinks and other beverages’ to investigate products in this category with HSR ≥ 2·5 that were classified as discretionary foods or UPF, as these products are not considered part of a healthy diet according to the ADG. ‘Other beverages’ in this category included cordials, hot chocolate, kombucha, fruit drinks and fruit juice syrups.

## Results

A total of 4724 food and beverage products displaying a HSR were released or updated between November 2020 and June 2023, representing 24·2 % of all products updated or released onto the Australian market during this period. Four variety pack items containing products with several different HSR were omitted, and the remaining 4720 products were included for further analysis. The median HSR for the total sample was 3·5 stars, and 77·5 % of all products analysed displayed a HSR equal to or higher than the pre-selected cut-off of 2·5 stars, considered a ‘healthy pass’ (Table 1). The most frequently displayed HSR was 4·0 stars, present on 21·7 % of all products analysed. Twenty-four different food and beverage categories were represented, with median HSR ranging from 0·5 for ‘Chocolate confectionery’, to 5·0 for ‘Juice drinks’.

**Table 1.** Descriptive statistics of Health Star Rating for total sample and by Mintel category

	HSR								
	*n*	%	Median	Mode	IQR	≤ 2·0		≥ 2·5	
						*n*	%	*n*	%
Total sample	4720	100	3·5	4·0	1·5	1060	22·5	3660	77·5
Mintel category	*n*	%	Median	Mode	IQR	≤ 2·0		≥ 2·5	
						*n*	%	*n*	%
Baby food	1	0·0	4·0	4·0	^*^	0	0·0	1	100
Bakery	765	16·2	2·0	3·5	2·0	391	51·1	374	48·9
Breakfast cereals	313	6·6	4·0	4·0	1·0	16	5·1	297	94·9
Carbonated soft drinks	64	1·4	3·5	3·5	3·0	29	45·3	35	54·7
Chocolate confectionery	56	1·2	0·5	0·5	0·4	53	94·6	3	5·4
Dairy	280	5·9	4·0	4·0	2·0	68	24·3	212	75·7
Desserts and ice cream	150	3·2	2·0	3·0	1·5	76	50·7	74	49·3
Fruit and vegetables	211	4·5	4·5	5·0	1·0	1	0·5	210	99·5
Hot beverages	3	0·1	1·0	0·5	^*^	2	66·6	1	33·3
Juice drinks	86	1·8	5·0	5·0	2·6	21	24·4	65	75·6
Meals and meal centres	504	10·7	3·5	3·5	1·0	33	6·5	471	93·5
Nutritional drinks and other beverages	73	1·5	5·0	5·0	0·5	7	9·6	66	90·4
Processed fish, meat and egg products	717	15·2	3·5	4·0	1·0	111	15·5	606	84·5
RTDs	25	0·5	2·5	3·5	1·5	7	28·0	18	72·0
Sauces and seasonings	245	5·2	3·0	4·0	2·0	64	26·1	181	73·9
Savoury spreads	22	0·5	3·5	3·5	1·1	5	22·7	17	77·2
Side dishes	220	4·7	4·0	4·0	0·5	1	0·5	219	99·5
Snacks	734	15·6	3·5	3·5	1·5	118	16·1	616	83·9
Soup	81	1·7	3·5	3·5	0·0	0	0·0	81	100
Sports and energy drinks	11	0·2	3·0	3·5	2·0	5	45·5	6	54·6
Sugar and gum confectionary	47	1·0	1·5	1·5	0·5	37	78·7	10	21·3
Sweet spreads	62	1·3	4·5	5·0	2·5	13	21·0	49	79·0
Sweeteners and sugar	6	0·1	3·5	3·5	3·0	2	33·3	4	66·7
Water	44	0·9	4·5	5·0	2·0	0	0·0	44	100

HSR, Health Star Rating; *n*, number of products; IQR, interquartile range; RTDs, ready-to-drink beverages.

* IQR not available due to sample size.

### Comparison of the Health Star Rating Scheme with the Nova Classification System

Most products in the sample were classified as UPF according to Nova (*n* 3671, 77·8 %), with MPF, PF and PCI comprising only 11·0 % (*n* 520), 10·6 % (*n* 499) and 0·6 % (*n* 30), respectively (Table 2). The median HSR was 4·5 stars for MPF, 4·0 stars for PF and 3·5 stars for both PCI and UPF. Distribution of HSR was skewed towards scores above the ‘healthy pass’ cut-off, with almost all MPF products (99 %) displaying HSR ≥ 2·5, and the majority of PF (84·8 %) and UPF (73·7 %) also displaying HSR equal to or above 2·5 stars. Only 26·3 % of UPF displayed a HSR ≤ 2·0. For all non-UPF products combined, the median HSR was 4·0 stars, which was significantly higher than the median HSR for UPF products (3·5 stars, *P* < 0·001). Cohen’s kappa showed that agreement between the HSR and Nova was none to slight (*k* = 0·09, *P* < 0·001).

**Table 2.** Descriptive statistics of Health Star Rating by Nova category

Nova category	HSR								
	*n*	%	Median	Mode	IQR	≤ 2·0		≥ 2·5	
						*n*	%	*n*	%
1 MPF	520	11·0	4·5	5·0	1·0	5	1	515	99
2 PCI	30	0·6	3·5	1·0	3·0	12	40	18	60
3 PF	499	10·6	4·0	4·0	1·5	76	15·2	423	84·8
4 UPF	3671	77·8	3·5	4·0	2·0	967	26·3	2704	73·7
	*n*	%	Median	Mode	IQR	≤ 2·0		≥ 2·5	
						*n*	%	*n*	%
Non-UPF Combined	1049	22·2	4·0^*^	5·0	1·5	93	8·8	956	91·2
UPF	3671	77·8	3·5	4·0	2·0	967	26·3	2704	73·7

HSR, Health Star Rating; *n*, number of products; IQR, interquartile range; MPF, Minimally processed foods; PCI, processed culinary ingredients; PF, processed foods; UPF, ultra-processed foods.

* Significantly higher than UPF median (Mann–Whitney U test, *P* < 0·001).

### Comparison of the Health Star Rating Scheme with the Australian Dietary Guidelines

A slightly higher number of products were classified as FFG products 53·1 % (*n* 2507), while discretionary foods comprised 46·9 % of all products (*n* 2213, Table 3). Most FFG products had HSR ≥ 2·5 (94·7 %, *n* 2374), and over half of discretionary foods (58·2 %, *n* 1286) also displayed HSR ≥ 2·5. The median HSR for all FFG products was 4·0, which was significantly higher than the median for discretionary foods (2·5 stars, *P* < 0·001). According to Cohen’s kappa, agreement between the HSR and ADG was fair (*k* = 0·38, *P* < 0·001).

**Table 3.** Descriptive statistics of Health Star Rating by Australian Dietary Guidelines category

Combined	HSR								
	*n*	%	Median	Mode	IQR	≤ 2·0		≥ 2·5	
						*n*	%	*n*	%
FFG	2507	53·1	4·0^*^	4·0	1·0	133	5·3	2374	94·7
Discretionary foods	2213	46·9	2·5	3·5	2·0	927	41·9	1286	58·1
ADG category	*n*	%	Median	Mode	IQR	≤ 2·0		≥ 2·5	
						*n*	%	*n*	%
Grains	688	14·6	4·0	4·0	1·0	25	3·6	663	96·3
Fruit	187	4·0	4·0	5·0	1·5	14	7·5	173	92·5
Vegetables/Legumes	302	6·4	4·5	5·0	1·0	0	0	302	100
Meat/Eggs/Tofu/Nuts/Seeds/Legumes	588	12·5	4·0	4·0	1·0	38	6·5	550	93·5
Milk/Cheese/Yoghurt/Alternatives	247	5·2	4·0	4·0	1·5	40	16·2	207	83·8
Mixed meals	437	9·3	3·5	3·5	1·5	10	2·3	427	97·7
Discretionary foods	2213	46·9	2·5	3·5	2·0	927	41·9	1286	58·1

ADG, Australian Dietary Guidelines; HSR, Health Star Rating; *n*, number of products; IQR, interquartile range; FFG, five food group foods.

* Significantly higher than discretionary foods median (Mann–Whitney U test, *P* < 0·001).

### Trends in Health Star Rating by year

**Figure 1.**
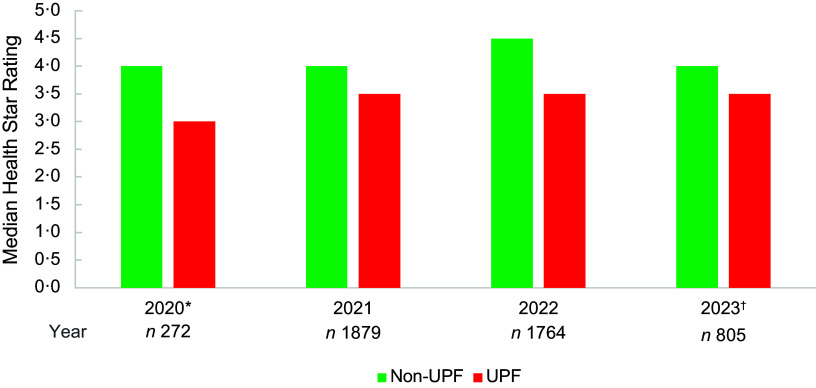
Median Health Star Rating by year for ultra-processed foods and non-ultra-processed foods. UPF, ultra-processed foods; Non-UPF, non-ultra-processed foods. *November–December 2020; ^†^January–June 2023.

The median HSR for the total sample was 3·5 stars for all years, except for the initial implementation year for the updated algorithm (3·0 stars), though this only included products released or updated from November 2020 (*n* 272, Figure 1). The proportion of products with HSR ≥ 2·5 ranged from 68·0 % in 2020, to 80·5 % in 2023 (online Supplementary Table 1).

Non-UPF products comprised 20·4–23·2 % of products released or updated each year, and UPF 76·8–79·6 % of products each year. The median HSR for non-UPF products was 4·0 stars for all years except 2022 (4·5 stars) and for UPF products 3·5 stars for all years except 2020 (3·0 stars). The proportion of UPF products displaying HSR ≥ 2·5 increased by year from 60·2 % in 2020 to 78·5 % in 2023 and decreased for non-UPF from 93·8 % in 2020 to 89·0 % in 2023.

**Figure 2.**
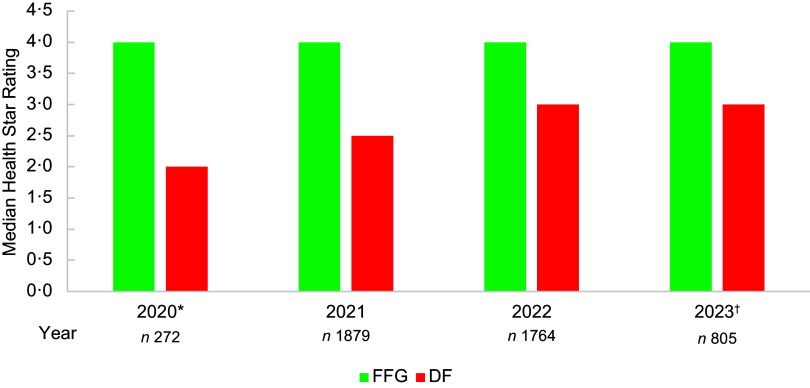
Median Health Star Rating by year for five food groups and discretionary food products. FFG, five food group foods; DF, discretionary foods. *November–December 2020; ^†^January–June 2023. Significant difference between median HSR for each year (Kruskal–Wallis test, *P* < 0·001).

FFG products comprised 50·7–55·3 % of products released or updated each year and discretionary foods 44·7–49·3 % of products each year. The median HSR for FFG products was 4·0 stars for all years, and for discretionary food products, the median was 2·0 and 2·5 stars in 2020 and 2021, respectively, and 3·0 stars in both 2022 and 2023 (Figure 2). The proportion of products displaying HSR ≥ 2·5 increased by year for both FFG and discretionary food products, from 88·3 % in 2020 to 95·0 % in 2023 for FFG and from 47·0 % in 2020 to 62·5 % in 2023 for discretionary foods. A Kruskal–Wallis test showed a statistically significant difference between median HSR for each year (*χ*^2^ (3, *n* 4720) = 22·49, *P* < 0·001), likely due to the increase in median for discretionary foods.

### Sub-analyses

The median HSR for ‘Juice drinks’ was 5·0 stars for all years from 2020 to 2022 and 2·3 stars for products released or updated to June 2023 (online Supplementary Table 2). The proportion of products with HSR > 3·5 (the maximum allowed HSR for fruit and vegetable juice drinks according to the updated HSR algorithm) varied over the implementation period from 72·4 % in 2021 (*n* 34) to 12·5 % in 2023 (*n* 1). When all juice drink products with HSR of 5·0 were adjusted to 3·5 stars to reflect the updated algorithm criteria for fruit juices, this reduced the median HSR to 3·5 for all years 2020–2022, while the median for 2023 remained unchanged at 2·3 (online Supplementary Table 3). After adjustment, the proportion of products with HSR > 3·5 ranged from 0 % in 2020 and 2023, to 7·3 % in 2022 (*n* 2).

The median HSR for ‘Fruit and vegetables’ was 4·5 stars for 2020 and 2021, 5·0 stars for 2022 and 4·8 stars for products released or updated to June 2023 (online Supplementary Table 2). The proportion of products with HSR of 5·0 increased each year, from 8·3 % in 2020 (*n* 1) to 59·3 % in 2022 (*n* 54). For the year to June 2023, the proportion of products with HSR of 5·0 was 50 % (*n* 21).

The median HSR for ‘Carbonated soft drinks’ was 3·5 stars, with 54·7 % (*n* 35) of these products displaying ‘healthy’ HSR of ≥ 2·5. These products are normally main sources of sugars and were also classified as discretionary foods and UPF. Further analyses revealed that 77·1 % (*n* 27) of these contained non-nutritive sweeteners (NNS), meaning they did not exceed sugar criteria that would require them to display a lower HSR (online Supplementary Table 4). ‘Sugars and sweeteners’ had a median HSR of 3·5, and 66·7 % of these products had HSR ≥ 2·5 (*n* 4). All products in this category were classified as discretionary foods and UPF but again all contained NNS, allowing them to display higher HSR. Conversely, although 79 % of ‘Sweet spreads’ (*n* 49) displayed HSR ≥ 2·5, several of these did not contain NNS but were nut butters, which were also included in this Mintel category. Of the products with HSR ≥ 2·5, 34·9 % contained added sugars, and five added-sugar products still received HSR of 5·0.

The majority of ‘Nutritional drinks and other beverages’ scored ≥ 2·5 stars (90·4 %, *n* 66), and the median was 5·0. These products included four cordials, and protein shakes, meal replacement shakes, liquid breakfast products, and weight loss shakes, as well as protein powder and other supplement powders (online Supplementary Table 5).

## Discussion

This study aimed to investigate changes in alignment between the HSR scheme, the Nova food processing classification system and the ADG during the period of implementation of adjustments to the HSR scheme’s algorithm. As predicted, a large proportion of discretionary foods and UPF were scored as ‘healthy’, with 58·2 % and 73·7 % of these products respectively displaying HSR ≥ 2·5 (out of 5), considered a ‘healthy pass’ score. The median HSR for UPF was 3·5 stars, indicating significant misalignment between the HSR and Nova. Food categories that are generally considered rich sources of sugar such as ‘Breakfast cereals’, ‘Carbonated soft drinks’, ‘Desserts and ice creams’, ‘Sports and energy drinks’, ‘Sweet spreads’, and ‘Sugars and sweeteners’ had greater proportions of products with high HSR. A higher proportion of UPF displayed HSR (*n* 3671, 77·8 % of all products) compared with non-UPF products, suggesting manufacturers’ selective application of the voluntary HSR scheme.

One of the main technical changes to the HSR algorithm was stricter criteria to increase the ‘penalty’ applied to sugar content. Most ‘Chocolate confectionery’ and ‘Sugar and gum confectionary’ products were scored HSR of ≤ 2·0 as expected; however, large proportions of other sweet food categories scored > 2·5 stars. Similar results were reported in an earlier study by Dickie *et al.* for ‘Chocolate confectionery’, ‘Sugar and gum confectionary’ and ‘Breakfast cereals’ (medians of 0·5, 1·5, and 4·0 stars, respectively)^(7)^. In contrast, the median for ‘Carbonated soft drinks’ was 3·5 stars compared with 1·5 stars in the previous study, and ‘Sweet spreads’ had a median of 4·5 stars compared with 4·0 stars^(7)^. This may be due to manufacturers reformulating their products with NNS and thus avoiding the tighter sugar scoring within the updated algorithm. Increased use of NNS has been widely observed in countries where nutrient profiling penalises sugar^(32)^ and is an unintended consequence of this approach which could have been mitigated under a scheme that also considers level of food processing^(28)^.

Manufacturers have also been able to obtain higher median HSR for many products in the category ‘Nutritional drinks and other beverages’ (nutritional drinks are classified as ‘special-purpose foods’ in the Australian and New Zealand Food Standards Code) by increasing the content of so-called beneficial components such as protein and fibre, while reducing sugar content by using NNS. This is an anomaly with the HSR, as most special-purpose foods are not permitted to display HSR, except for formulated meal replacements and formulated supplementary foods, provided they conform to criteria set out in the Food Standards Code Standard 2.9.3^(9,33)^. This illustrates the need for the HSR algorithm to assess degree of processing, as the ADG do not recommend these products as part of a healthy diet, and they are UPF.

Other updates to the HSR criteria included a reduced upper limit for sodium; redefinition of dairy products to include custards, evaporated milks, dairy-based desserts, cream, crème fraiche, sour cream, cream cheeses and mascarpone; automatic 5-star ratings for minimally processed fruit and vegetables and plain water; and automatic 4·5-star ratings for unsweetened flavoured waters. Compared with previous research which reported a median HSR of 2·0 for ‘Water’,^(7)^ the current study showed an increase to 4·5 stars and a slight decrease in median HSR for ‘Snacks’ from 4·0 in the previous study^(7)^ to 3·5 in the current study, possibly due to increased sensitivity to sugar and salt under the updated criteria. The proportion of ‘Fruit and vegetables’ displaying 5 stars increased between 2020 and 2022; however, there was no change in median HSR for this category despite all minimally processed produce automatically receiving 5 stars under the new algorithm. This may reflect the inclusion of canned fruit, vegetables and legume products with added salt or sugar in the ‘Fruit and vegetables’ category in Mintel.

Although the HSR aligned well with Nova’s non-UPF categories (91·2 % scored ≥ 2·5 stars), this was not the case for UPF products, as 73·7 % of these displayed a score over the ‘healthy’ cut-off, similar to results reported for the original HSR algorithm (73 % ≥ 2·5 stars)^(7)^. The median HSR for UPF was the same in both the previous and current studies (3·5 stars), which is unsurprising given the update did not incorporate any elements of level of processing. Similar findings have been reported from the evaluation of the recently updated European Nutri-Score scheme that had adjusted nutrient criteria, food category definitions and cut-off thresholds, which is underpinned by a nutrient profiling model comparable to that used by the HSR^(34)^. Studies have found an overrepresentation of UPF in ‘healthy’ Nutri-Score categories, with one study reporting that 71·5 % of Nutri-Score A products were UPF^(35)^, and another finding that 89 % of products scoring A or B were UPF^(36)^. This suggests that altering nutrient criteria alone may be insufficient and that the problem of UPF being labelled as healthy by FOPL may instead arise from dissonance between the nutrient-specific paradigm underpinning these models and the holistic, food-based concept upon which Nova is based.

The HSR also aligned closely with the ADG for healthy foods, and although a greater proportion of unhealthy foods were awarded HSR ≤ 2·0 than in the comparison with Nova, 58·1 % of all discretionary foods still displayed HSR over the ‘healthy’ cut-off. Similar results were reported by Dickie *et al.*, with 52·8 % of discretionary food products scoring ≥ 2·5 stars, and the median HSR for discretionary foods was also the same as the current study (2·5 stars)^(7)^. This suggests that the algorithm changes may not have managed to align the HSR with the ADG.

The number of products released or updated each year was similar for all complete years included in analyses, though there was a trend towards an increased proportion of products scoring HSR ≥ 2·5 each year, and a corresponding increase in the proportions of UPF and discretionary foods scoring ≥ 2·5 stars. The median HSR for UPF was relatively stable over this period, but gradually increased for discretionary foods. This is concerning considering previous research has already reported an overrepresentation of unhealthy (UPF or discretionary food) products displaying HSR and that higher HSR are more frequently displayed under the current system^(7,19,20)^. This can give UPF and discretionary foods a ‘health halo’, making them appear healthier than non-UPF and FFG foods that do not display HSR, and may influence consumer purchases negatively. Furthermore, most UPF and discretionary foods are packaged, enabling the HSR to be more readily applied to these foods than to non-UPF and FFG foods. Selective application of the HSR is a weakness of the voluntary system, and many recommend a mandatory scheme^(23,37)^. However, mandating the implementation of the HSR will not address its two core design flaws that have resulted in it frequently being exploited as a marketing tool for promoting UPF. First, the HSR algorithm is able to be consistently manipulated by UPF manufacturers to inflate the calculation of their product’s HSR scores, for example, by substituting industrial ingredients such as NNS for so-called ‘risk’ nutrients, such as added sugar, and/or adding industrial ingredients such as protein isolates and fibre powders to claim higher levels of so-called ‘beneficial’ nutrients^(19)^. Second, the algorithm does not differentiate whether a food product is a nutritious food or an UPF, with consumers often being encouraged to substitute one UPF for another UPF, a process that Bennett *et al.* have reported consumers find unhelpful, confusing and misleading^(24)^.

The two-year implementation period for compliance with the updated algorithm may have allowed manufacturers to exploit a ‘health halo’ effect for certain products by maximising the full transition period rather than implementing changes earlier. This is particularly evident in the analyses of ‘Juice drinks’, which had a median of 5·0 stars for 2020–2022, then dropped to 2·3 stars for 2023. Conversely, analyses of ‘Fruit and vegetables’ showed a slight trend towards earlier adoption of new criteria, which allowed higher HSR for this category. As each product entered into Mintel (including updated existing products) is assigned a new Record ID, it was not possible within the scope of this study to determine how many of these products have altered HSR due to the new algorithm.

Considerable resources were allocated to updating the HSR, which has produced some improvements in alignment with the Nova system and ADG over the implementation period for the updated algorithm^(7)^. Although criteria related to risk nutrients such as sugar and sodium were altered in the new algorithm, food categories that are the main sources of these had greater proportions of products with high HSR in the current study, incorrectly classifying these products as ‘healthy’. As reported in previous research, there is still an overrepresentation of UPF products displaying HSR amongst newly released products^(7,19,20)^ and high HSR are being awarded to a large proportion of UPF and to just over half of discretionary foods. This could suggest that further changes are required to significantly improve scoring of unhealthy food products in line with Nova and the ADG. Similarities between this study and previous research also indicate that it may not be possible to adjust existing criteria to adequately account for UPF and discretionary foods, as the HSR is based upon reductionist nutrient-specific criteria which does not consider level or purpose of processing when assessing a food’s healthiness. Instead, an integrated FOPL model that first considers degree and purpose of processing, then profiles the presence of risk and beneficial nutrients has been recommended^(28)^. A novel FOPL incorporating these criteria was recently modelled by Dickie *et al.* ^(28)^ and when compared with the HSR classified a higher proportion of food categories in line with healthy dietary patterns, as well as those consistent with adverse health outcomes.

### Strengths and limitations

Although the Mintel Global New Products Database is considered comprehensive, data are collected by shoppers rather than trained researchers, and accuracy of data is therefore reliant upon input of correct HSR and product data by Mintel shoppers. Although a large number of products are identified each month, the actual number of new products that are released into the market is unknown. Additionally, product launch dates recorded in Mintel may not be precise, which limits the ability to evaluate alignment between the updated HSR, Nova and the ADG. Some of the results of this study therefore may not necessarily be attributable to the algorithm update.

As Mintel only collects information on products flagged as new or updated, and many new UPF or discretionary food products or flavours tend to be snack foods, this may have influenced results towards a higher proportion of UPF and discretionary foods.

Many new food products tend to be discretionary, and some products present in the current marketplace could not be matched to items in the Discretionary Food List developed by the Australian Bureau of Statistics for the 2011–2013 Australian Health Survey. An updated Discretionary Food List was developed for use in the 2023 National Nutrition and Physical Activity Survey^(38)^, and had this been available at the time the current research was undertaken, the results have shown a modest difference in the proportion of discretionary foods with ‘healthy’ HSR. The classification process related to Nova may have resulted in an underestimation of UPF in the sample, as criteria used in this and related earlier studies rely on presence of MUP to identify UPF^(7,28)^ and do not consider industrial processing methods used in the manufacture of certain products.

Finally, although the same database and similar methods were used in a previous study by Dickie *et al.* ^(7)^, actual products included in analyses differ between this and the current study. This is due to data collection being restricted to products released in a specific period for each study, which produced differences in sample size and number of products in each food category. Furthermore, limitations in the scope of this study meant it was not possible to identify UPF or discretionary food products with low HSR that may have stopped displaying HSR or those without HSR that started displaying high HSR during the study period.

Food group comparisons with previous research evaluating the original HSR algorithm were not conducted. This is an important area for future research to identify specific food categories where the algorithm is awarding HSR that are misaligned with the Nova and ADG ratings. Additionally, analyses with re-coded product items according to the updated Australian Bureau of Statistics Discretionary Food List would increase the accuracy of the (mis)alignment between the HSR and the ADG based upon the current food supply.

### Conclusion

The HSR classified a significant proportion of UPF and discretionary foods as ‘healthy’, awarding these products scores at or above a ‘healthy pass’ cut-off of ≥ 2·5 stars. This finding is consistent with that reported in an earlier study investigating the HSR scheme prior to the implementation period for the updated algorithm. The results of the current study suggest that recent updates to the HSR scheme algorithm may not have substantially improved its alignment with the ADG and Nova. This persistent lack of alignment raises ongoing doubts about the ability of the HSR’s nutrient-based approach to translate food-and diet-based nutrition policy recommendations into accurate food ‘healthiness’ assessments when designing nutrition policy interventions.

## Supporting information

10.1017/S1368980026102419.sm001 Brastein et al. supplementary material Brastein et al. supplementary material

## Supplementary material

For supplementary material accompanying this paper visit https://doi.org/10.1017/S1368980026102419
