# Supplementary material for: An investigation of the change in alignment between the Health Star Rating Scheme and the Nova Food Processing Classification System and the Australian Dietary Guidelines
Source: Public Health Nutr. 2026 Mar 27;29(1):e82. doi: 10.1017/S1368980026102419 (PMC13087985; doi:10.1017/S1368980026102419)
Supplement: Brastein et al. supplementary material [file S1368980026102419sup001.docx]

**Supplementary Table 1: Descriptive Statistics of Health Star Rating by Year for Total Sample, and by Nova and Australian Dietary Guidelines Category**

| **Total Sample** |  |  |  | **HSR** |  |  |  |
| --- | --- | --- | --- | --- | --- | --- | --- |
| **Year** | **n** | **%** | **Median** | **Mode** | **IQR** | **n (%) ≤2.0** | **n (%) ≥2.5** |
| 2020* | 272 | 100 | 3.0 | 3.5 | 2.0 | 87 (32.0) | 185 (68.0) |
| 2021 | 1879 | 100 | 3.5 | 4.0 | 1.5 | 430 (22.9) | 1449 (77.1) |
| 2022 | 1764 | 100 | 3.5 | 4.0 | 1.5 | 386 (21.9) | 1378 (78.1) |
| 2023** | 805 | 100 | 3.5 | 3.5 | 1.5 | 157 (19.5) | 648 (80.5) |
|  |  |  |  |  |  |  |  |
| **Non-UPF** | **n** | **%** | **Median** | **Mode** | **IQR** | **n (%) ≤2.0** | **n (%) ≥2.5** |
| 2020* | 63 | 23.2 | 4.0 | 4.0 | 1.0 | 4 (6.3) | 59 (93.7) |
| 2021 | 413 | 22 | 4.0 | 5.0 | 1.5 | 34 (8.2) | 379 (91.8) |
| 2022 | 409 | 23.2 | 4.5 | 5.0 | 1.5 | 37 (9.0) | 372 (90.9) |
| 2023** | 164 | 20.4 | 4.0 | 4.0 | 1.5 | 18 (11.0) | 146 (89.0) |
|  |  |  |  |  |  |  |  |
| **UPF** |  |  |  |  |  |  |  |
| 2020* | 209 | 76.8 | 3.0 | 3.5 | 2.0 | 83 (39.7) | 126 (60.3) |
| 2021 | 1466 | 78 | 3.5 | 4.0 | 2.0 | 397 (27.0) | 1070 (73.0) |
| 2022 | 1355 | 76.8 | 3.5 | 4.0 | 2.0 | 349 (25.8) | 1006 (74.2) |
| 2023** | 641 | 79.6 | 3.5 | 3.5 | 1.5 | 139 (21.7) | 502 (78.3) |
|  |  |  |  |  |  |  |  |
| **FFG** | **n** | **%** | **Median** | **Mode** | **IQR** | **n (%) ≤2.0** | **n (%) ≥2.5** |
| 2020* | 138 | 50.7 | 4.0 | 4.0 | 1.0 | 16 (11.6) | 122 (88.4) |
| 2021 | 997 | 53.1 | 4.0 | 4.0 | 1.0 | 48 (4.8) | 949 (95.2) |
| 2022 | 927 | 52.6 | 4.0 | 4.0 | 1.0 | 47 (5.1) | 880 (94.9) |
| 2023** | 445 | 55.3 | 4.0 | 4.0 | 1.0 | 22 (4.9) | 423 (95.1) |
|  |  |  |  |  |  |  |  |
| **DF** |  |  |  |  |  |  |  |
| 2020* | 134 | 49.3 | 2.0 | 1.5 | 1.5 | 71 (53.0) | 63 (47.0) |
| 2021 | 882 | 46.9 | 2.5 | 3.0 | 2.0 | 382 (43.3) | 500 (56.7) |
| 2022 | 837 | 47.4 | 3.0 | 3.5 | 2.0 | 339 (40.5) | 498 (59.5) |
| 2023** | 360 | 44.7 | 3.0 | 3.5 | 2.0 | 135 (37.5) | 225 (62.5) |
|  |  |  |  |  |  |  |  |

*HSR: Health Star Rating; n: number of products; IQR: interquartile range; UPF: Ultra-Processed Foods; ADG: Australian Dietary Guidelines; FFG: Five Food Group foods; DF: Discretionary Foods*

** November – December 2020
** January – June 2023*

**Supplementary Table 2: Descriptive Statistics of Health Star Rating by Year for Juice Drinks and Fruit and Vegetables**

| **Juice Drinks** |  |  | **HSR** | | |
| --- | --- | --- | --- | --- | --- |
| **Year** | **n** | **%** | **Median** | **n (%) ≤3.5** | **n (%) >3.5** |
| 2020* | 3 | 100 | 5.0 | 1 (33.3) | 2 (66.7) |
| 2021 | 47 | 100 | 5.0 | 13 (27.7) | 34 (72.4) |
| 2022 | 28 | 100 | 5.0 | 11 (39.2) | 17 (60.7) |
| 2023** | 8 | 100 | 2.3 | 7 (87.5) | 1 (12.5) |
|  |  |  |  |  |  |
| **Fruit and Vegetables** |  |  | **HSR** | | |
| **Year** | **n** | **%** | **Median** | **n (%) ≤4.5** | **n (%) 5.0** |
| 2020* | 12 | 100 | 4.5 | 11 (91.6) | 1 (8.3) |
| 2021 | 66 | 100 | 4.5 | 40 (60.6) | 26 (39.4) |
| 2022 | 91 | 100 | 5.0 | 37 (40.7) | 54 (59.3) |
| 2023** | 42 | 100 | 4.8 | 21 (50.1) | 21 (50) |
|  |  |  |  |  |  |

*HSR: Health Star Rating; n: number of products*

** November – December 2020
** January – June 2023*

**Supplementary Table 3: Adjusted Health Star Rating by Year for Juice Drinks**

| **Juice Drinks** |  |  | **HSR#** | | |
| --- | --- | --- | --- | --- | --- |
| **Year** | **n** | **%** | **Median** | **n (%) ≤3.5** | **n (%) >3.5** |
| 2020* | 3 | 100 | 3.5 | 3 (100) | 0 (0) |
| 2021 | 47 | 100 | 3.5 | 45 (95.8) | 2 (4.2) |
| 2022 | 28 | 100 | 3.5 | 26 (92.7) | 2 (7.3) |
| 2023** | 8 | 100 | 2.3 | 8 (100) | 0 (0) |
|  |  |  |  |  |  |

*HSR: Health Star Rating; n: number of products*

# *HSR for all Juice Drinks scoring 5.0 was adjusted to 3.5 for this analysis*

** November – December 2020
** January – June 2023*

**Supplementary Table 4: Carbonated Soft Drinks, Sweet Spreads, and Sugars and Sweeteners with Health Star Rating ≥2.5**

| **Carbonated Soft Drinks**  **(n=35)** | |  |  | |  | | |  |  |  |  |  |
| --- | --- | --- | --- | --- | --- | --- | --- | --- | --- | --- | --- | --- |
| **Product Name** | | | | **HSR** | **ADGs** | **NOVA** | | **NNS** |  |  |  |  |
| Coca-Cola No Sugar | | | | 3.5 | 7 | 4 | | Y |  |  |  |  |
| Coca-Cola No Sugar | | | | 3.5 | 7 | 4 | | Y |  |  |  |  |
| Coca-Cola No Sugar No Caffeine | | | | 3.5 | 7 | 4 | | Y |  |  |  |  |
| Coca-Cola No Sugar Vanilla Flavour | | | | 3.5 | 7 | 4 | | Y |  |  |  |  |
| Coles No Sugar Dry Ginger Ale Sparkling Mixer | | | | 3.5 | 7 | 4 | | Y |  |  |  |  |
| Coles No Sugar Lemon Flavour | | | | 4.0 | 7 | 4 | | Y |  |  |  |  |
| Coles No Sugar Raspberry Flavour | | | | 4.0 | 7 | 4 | | Y |  |  |  |  |
| Coles No Sugar Tonic Water Sparkling Mixer | | | | 3.5 | 7 | 4 | | Y |  |  |  |  |
| Diet Coca-Cola | | | | 3.5 | 7 | 4 | | Y |  |  |  |  |
| Diet Coca-Cola | | | | 3.5 | 7 | 4 | | Y |  |  |  |  |
| Diet Tonic Water | | | | 3.5 | 7 | 4 | | Y |  |  |  |  |
| Fanta #What the Fanta? | | | | 3.5 | 7 | 4 | | Y |  |  |  |  |
| Fanta Mini No Sugar Raspberry | | | | 3.5 | 7 | 4 | | Y |  |  |  |  |
| Fanta No Sugar Orange Flavour | | | | 3.5 | 7 | 4 | | Y |  |  |  |  |
| Fanta No Sugar Orange Flavour | | | | 3.5 | 7 | 4 | | Y |  |  |  |  |
| Fanta No Sugar Raspberry Flavour | | | | 3.5 | 7 | 4 | | Y |  |  |  |  |
| Kirks Sugar Free Pasito Passionfruit Flavour | | | | 3.5 | 7 | 4 | | Y |  |  |  |  |
| Lemon + Zero Sugar Sprite | | | | 3.5 | 7 | 4 | | Y |  |  |  |  |
| Lemon Pepsi Max | | | | 3.5 | 7 | 4 | | Y |  |  |  |  |
| Lemon-Lime Natural Sprite No Sugar | | | | 3.0 | 7 | 4 | | Y |  |  |  |  |
| Lemon-Lime Sprite No Sugar | | | | 3.5 | 7 | 4 | | Y |  |  |  |  |
| Lemon-Lime Sprite No Sugar | | | | 3.5 | 7 | 4 | | Y |  |  |  |  |
| Lemon-Lime Sprite Zero | | | | 3.5 | 7 | 4 | | Y |  |  |  |  |
| Mildura No Added Sugar Sparkling Apple, Lemon & Lime Fruit Drink | | | | 2.5 | 7 | 4 | | N |  |  |  |  |
| Pepsi Max | | | | 3.5 | 7 | 4 | | Y |  |  |  |  |
| Solo Zero Sugar Original Lemon Flavour Thirst Crusher | | | | 3.5 | 7 | 4 | | Y |  |  |  |  |
| Sprite No Sugar Lemon-Lime Natural Flavoured Soft Drink | | | | 3.0 | 7 | 4 | | Y |  |  |  |  |
| Sunkist Zero Sugar Orange Flavour | | | | 3.5 | 7 | 4 | | Y |  |  |  |  |
| The Good Pear Sparkling Pear Juice | | | | 5.0 | 7 | 4 | | N |  |  |  |  |
| Vitamelon Sparkling Pressed Sparkling Watermelon and Peach Juice | | | | 4.0 | 7 | 4 | | N |  |  |  |  |
| Vitamelon Sparkling Pressed Sparkling Watermelon Juice | | | | 4.0 | 7 | 4 | | N |  |  |  |  |
| Vitamelon Sparkling Pressed Sparkling Watermelon Juice with Pineapple | | | | 4.0 | 7 | 4 | | N |  |  |  |  |
| Vitamelon Sparkling Sparkling Watermelon Juice | | | | 4.0 | 7 | 4 | | N |  |  |  |  |
| Vitamelon Sparkling Sparkling Watermelon Juice with Peach | | | | 4.0 | 7 | 4 | | N |  |  |  |  |
| Vitamelon Sparkling Sparkling Watermelon Juice with Pineapple | | | | 4.0 | 7 | 4 | | N |  |  |  |  |
| **Sweet Spreads**  **(n=49)** |  | | | |  | |  |  |  |  |  |  |
| \| **Product Name** \| **HSR** \| **ADGs** \| **NOVA** \| **NNS** \| \| --- \| --- \| --- \| --- \| --- \| \| Bramwells American Style Crunchy Peanut Butter \| 4.0 \| 4 \| 4 \| N \| \| Buddee Chocolate Spread \| 4.5 \| 7 \| 4 \| N \| \| Buddee Crunchy Spread \| 5.0 \| 4 \| 4 \| N \| \| Buddee Smooth Spread \| 5.0 \| 4 \| 4 \| N \| \| Coles Crunchy Peanut Butter \| 4.0 \| 4 \| 4 \| N \| \| Coles Organic Crunchy Peanut Butter \| 4.5 \| 4 \| 1 \| N \| \| Coles Organic Crunchy Peanut Butter \| 5.0 \| 4 \| 1 \| N \| \| Coles Organic Smooth Peanut Butter \| 4.5 \| 4 \| 1 \| N \| \| Coles Smooth Choc Peanut Butter \| 2.5 \| 7 \| 4 \| N \| \| Coles Smooth Peanut Butter \| 4.0 \| 4 \| 4 \| N \| \| Coles Wellness Road Cashew Butter \| 5.0 \| 4 \| 3 \| N \| \| Coles Wellness Road Super Nutty 3 Nut Spread \| 5.0 \| 4 \| 3 \| N \| \| Fix & Fogg Peanut Butter and Jelly \| 4.0 \| 7 \| 4 \| N \| \| Honest to Goodness Organic Crunchy Peanut Butter \| 5.0 \| 4 \| 1 \| N \| \| Just Organic Raspberry Spread \| 2.5 \| 7 \| 4 \| N \| \| Just Organic Strawberry Spread \| 2.5 \| 7 \| 4 \| N \| \| Lakanto No Added Sugar Bakers Vanilla Syrup with Monkfruit Sweetener \| 3.5 \| 7 \| 4 \| Y \| \| Lakanto No Added Sugar Golden Malt Flavoured Syrup with Monkfruit Sweetener \| 3.5 \| 7 \| 4 \| Y \| \| Macro Wholefoods Market Cashew Spread \| 4.0 \| 4 \| 1 \| N \| \| Macro Wholefoods Market Raspberry Spread \| 2.5 \| 7 \| 4 \| N \| \| Mayver's Crunchy Peanut Butter \| 5.0 \| 4 \| 3 \| N \| \| Mayver's Crunchy Peanut Butter \| 5.0 \| 4 \| 3 \| N \| \| Mayver's Dark Roasted Crunchy Peanut Butter \| 5.0 \| 4 \| 3 \| N \| \| Mayver's Dark Roasted Extra Crunchy Peanut Butter \| 5.0 \| 4 \| 3 \| N \| \| Mayver's Extra Crunchy Peanut Butter \| 5.0 \| 4 \| 3 \| N \| \| Mayver's Manuka Peanut Butter \| 4.5 \| 4 \| 4 \| N \| \| Mayver's Omega 3 Super Peanut Butter \| 5.0 \| 4 \| 3 \| N \| \| Mayver's Probiotic Super Peanut Butter \| 5.0 \| 4 \| 4 \| N \| \| Mayver's Skin On Smunchy Peanut Butter \| 5.0 \| 4 \| 3 \| N \| \| Mayver's Smooth Hazelnut Cashew & Cacao Butter \| 5.0 \| 7 \| 3 \| N \| \| Mayver's Smooth Peanut Butter \| 5.0 \| 4 \| 3 \| N \| \| Mayver's Smooth Peanut Butter \| 5.0 \| 4 \| 3 \| N \| \| Mayver's Smooth Peanut Spread \| 5.0 \| 4 \| 1 \| N \| \| Mayver's Smunchy Almond Butter \| 5.0 \| 4 \| 3 \| N \| \| Mayver's Smunchy Fibre + Peanut Butter \| 5.0 \| 4 \| 4 \| N \| \| Mayver's Super Collagen Peanut Butter \| 4.5 \| 4 \| 4 \| N \| \| Natvia Pantry Range 95% Less Sugar Strawberry Fruit Spread \| 4.5 \| 7 \| 4 \| Y \| \| Natvia Raspberry Fruit Spread with Chia Seeds \| 5.0 \| 7 \| 4 \| Y \| \| Natvia Strawberry Fruit Spread with Chia Seeds \| 4.0 \| 7 \| 4 \| Y \| \| Oh So Natural Wholefoods Almond Spread \| 5.0 \| 4 \| 1 \| N \| \| Oh So Natural Wholefoods Cashew Spread \| 5.0 \| 4 \| 1 \| N \| \| Oh So Natural Wholefoods Crunchy Peanut Butter \| 5.0 \| 4 \| 1 \| N \| \| Oh So Natural Wholefoods Smooth Peanut Butter \| 5.0 \| 4 \| 1 \| N \| \| Sanitarium Natural Crunchy Peanut Butter \| 5.0 \| 4 \| 1 \| N \| \| Sanitarium Nutilicious Smooth Peanut Butter \| 3.0 \| 4 \| 3 \| N \| \| The No Nasties Project 50% Less Sugar Choc Hazelnut Spread \| 3.0 \| 7 \| 4 \| N \| \| VGood Crunchy PeaNot Butter \| 5.0 \| 7 \| 3 \| N \| \| VGood Hazelnut Choc Spread \| 5.0 \| 7 \| 4 \| N \| \| VGood Smooth PeaNot Butter \| 5.0 \| 7 \| 3 \| N \| | | | | | | | | | |  |  |  |

| **Sweeteners and Sugar**  **(n=4)** | |  |  | |  | | | | |  |
| --- | --- | --- | --- | --- | --- | --- | --- | --- | --- | --- |
| **Product Name** | | | | **HSR** | | **ADGs** | | **NOVA** | | **NNS** |
| FreshLife Monk Fruit Sweetener with Erythritol | | | | 3.5 | | 7 | | 4 | | Y |
| Lakanto 99% Sugar-Free Monkfruit Sweetener Baking Blend with Classic Sweetener | | | | 5.0 | | 7 | | 4 | | Y |
| Lakanto Classic Monkfruit Sweetener | | | | 3.5 | | 7 | | 4 | | Y |
| Lakanto Sugar-Free Monkfruit Sweetener Icing Powder | | | | 3.5 | | 7 | | 4 | | Y |
|  |  | | | | | |  | |  |  |

** Duplicates are products with different unit sizes or repeated product updates over the data collection period*

**Supplementary Table 5: Nutritional Drinks and Other Beverages with Health Star Rating ≥2.5**

| **Nutritional Drinks and Other Beverages**  **(n=66)** |  |  |  |  |  |  |  |
| --- | --- | --- | --- | --- | --- | --- | --- |
| \| **Product Name** \| \| **HSR** \| **ADGs** \| \| **NOVA** \| \| \| --- \| --- \| --- \| --- \| --- \| --- \| --- \| \| Atkins Creamy Vanilla Low Carb Protein Shake \| \| 5.0 \| 7 \| \| 4 \| \| \| Atkins Low Carb Vanilla Flavoured Protein Shake Mix \| \| 3.5 \| 7 \| \| 4 \| \| \| Coles BOM Banana Flavour Liquid Breakfast On The Move \| \| 5.0 \| 7 \| \| 4 \| \| \| Coles BOM Caramel Flavour Liquid Breakfast On The Move \| \| 5.0 \| 7 \| \| 4 \| \| \| Coles BOM Chocolate Flavour Dairy Free Oat Milk Breakfast On The Move \| \| 5.0 \| 7 \| \| 4 \| \| \| Coles BOM Chocolate Flavour Liquid Breakfast On The Move \| \| 5.0 \| 7 \| \| 4 \| \| \| Coles BOM Coffee Flavour Liquid Breakfast On The Move \| \| 5.0 \| 7 \| \| 4 \| \| \| Coles BOM Strawberry Flavour Liquid Breakfast On The Move \| \| 5.0 \| 7 \| \| 4 \| \| \| Coles BOM Vanilla Flavour Liquid Breakfast On The Move \| \| 5.0 \| 7 \| \| 4 \| \| \| Coles Lime Cordial \| \| 2.5 \| 7 \| \| 4 \| \| \| Coles Wellness Road Australian Faba Bean Protein Powder \| \| 4.0 \| 7 \| \| 1 \| \| \| Fine Fettle Veg Extra Eat the Rainbow Vegetable Powder \| \| 5.0 \| 3 \| \| 1 \| \| \| Fine Fettle Veg Extra Good Gut Powder \| \| 5.0 \| 3 \| \| 1 \| \| \| Fine Fettle Veg Extra Mighty Root Veg Powder \| \| 4.5 \| 3 \| \| 1 \| \| \| Fine Fettle Veg Extra Mood Booster Drink Powder \| \| 5.0 \| 3 \| \| 1 \| \| \| Isowhey Assorted Flavour Men's Shake Formulated Meal Replacement \| \| 5.0 \| 7 \| \| 4 \| \| \| Isowhey Chocolate Caramel Flavour Men's Shake Formulated Meal Replacement \| \| 5.0 \| 7 \| \| 4 \| \| \| Isowhey Chocolate Flavour Men's Shake Formulated Meal Replacement \| \| 5.0 \| 7 \| \| 4 \| \| \| Isowhey Chocolate Fudge VLCD Shake + Collagen \| \| 5.0 \| 7 \| \| 4 \| \| \| Isowhey Cookies 'N Cream Flavour Men's Shake Formulated Meal Replacement \| \| 5.0 \| 7 \| \| 4 \| \| \| Isowhey Espresso Optimum VLCD Shake + Collagen \| \| 5.0 \| 7 \| \| 4 \| \| \| Isowhey French Vanilla Optimum VLCD Shake + Collagen \| \| 5.0 \| 7 \| \| 4 \| \| \| Isowhey Strawberries & Cream Flavour Optimum VLCD Shake + Collagen \| \| 5.0 \| 7 \| \| 4 \| \| \| Isowhey Vanilla Flavour Men's Shake Formulated Meal Replacement \| \| 5.0 \| 7 \| \| 4 \| \| \| Kapowder Enhance Collagen Booster for Hair and Nails Dietary Supplement Powder \| \| 5.0 \| 7 \| \| 4 \| \| \| Naturopathica FatBlaster Chocolate Flavour Weight Loss Shake for Men \| \| 5.0 \| 7 \| \| 4 \| \| \| Naturopathica FatBlaster Platinum+ ThermoBurn Chocolate Flavour Weight Loss Shake \| \| 5.0 \| 7 \| \| 4 \| \| \| Naturopathica FatBlaster Platinum+ ThermoBurn Chocolate Flavour Weight Loss Shake \| \| 5.0 \| 7 \| \| 4 \| \| \| Naturopathica FatBlaster Platinum+ ThermoBurn Coffee Flavour Weight Loss Shake \| \| 5.0 \| 7 \| \| 4 \| \| \| Naturopathica FatBlaster Platinum+ ThermoBurn Coffee Flavour Weight Loss Shake \| \| 5.0 \| 7 \| \| 4 \| \| \| Naturopathica FatBlaster Platinum+ ThermoBurn Vanilla Flavour Weight Loss Shake \| \| 5.0 \| 7 \| \| 4 \| \| \| Naturopathica FatBlaster Platinum+ ThermoBurn Vanilla Flavour Weight Loss Shake \| \| 5.0 \| 7 \| \| 4 \| \| \| Natvia Apple Raspberry Cordial Concentrate \| \| 3.0 \| 7 \| \| 4 \| \| \| Natvia Blood Orange & Lime Cordial Concentrate \| \| 3.0 \| 7 \| \| 4 \| \| \| Natvia Tropical Cordial Concentrate \| \| 3.0 \| 7 \| \| 4 \| \| \| Nexba Chocolate Liquid Brekkie \| \| 5.0 \| 7 \| \| 4 \| \| \| Nexba Coffee Liquid Brekkie \| \| 5.0 \| 7 \| \| 4 \| \| \| Nexba Rich Chocolate Liquid Brekkie \| \| 4.5 \| 7 \| \| 4 \| \| \| Nexba Strawberry Liquid Brekkie \| \| 5.0 \| 7 \| \| 4 \| \| \| Nexba Strawberry Liquid Brekkie \| \| 4.5 \| 7 \| \| 4 \| \| \| Nexba Vanilla Liquid Brekkie \| \| 5.0 \| 7 \| \| 4 \| \| \| Nexba Vanilla Liquid Brekkie \| \| 4.5 \| 7 \| \| 4 \| \| \| Rapid Wellness Rapid Loss Banana Flavour Meal Replacement Shake \| \| 4.0 \| 7 \| \| 4 \| \| \| Rapid Wellness Rapid Loss Chocolate Flavour Meal Replacement Shake \| \| 4.0 \| 7 \| \| 4 \| \| \| Rapid Wellness Rapid Loss Salted Caramel Flavour Meal Replacement Shake \| \| 3.5 \| 7 \| \| 4 \| \| \| Rapid Wellness Rapid Loss Vanilla Flavour Meal Replacement Shake \| \| 5.0 \| 7 \| \| 4 \| \| \| Sanitarium Up & Go Choc Ice Flavoured Liquid Breakfast \| \| 4.5 \| 7 \| \| 4 \| \| \| Sanitarium Up & Go Dairy Free Choc Ice Flavoured Liquid Breakfast \| \| 5.0 \| 7 \| \| 4 \| \| \| Sanitarium Up & Go Dairy Free Choc Ice Flavoured Liquid Breakfast \| \| 5.0 \| 7 \| \| 4 \| \| \| Sanitarium Up & Go Dairy Free Vanilla Ice Flavoured Liquid Breakfast \| \| 5.0 \| 7 \| \| 4 \| \| \| Sanitarium Up & Go Dairy Free Vanilla Ice Flavoured Liquid Breakfast \| \| 3.5 \| 7 \| \| 4 \| \| \| Sanitarium Up & Go Protein Energize Choc Hit Flavour Formulated Supplementary Food \| \| 5.0 \| 7 \| \| 4 \| \| \| Sanitarium Up & Go Protein Energize Iced Coffee Flavoured Protein Beverage \| \| 5.0 \| 7 \| \| 4 \| \| \| Sanitarium Up & Go Strawberry Flavoured Protein Drink \| \| 5.0 \| 7 \| \| 4 \| \| \| The Healthy Mummy Caramel Flavour Premium Blend Smoothie \| \| 5.0 \| 7 \| \| 4 \| \| \| The Healthy Mummy Double Chocolate Premium Blend Smoothie \| \| 5.0 \| 7 \| \| 4 \| \| \| The Healthy Mummy Vanilla Flavour Pregnancy Smoothie \| \| 5.0 \| 7 \| \| 4 \| \| \| The Healthy Mummy Vanilla Flavour Weight Loss Smoothie Premium Blend \| \| 5.0 \| 7 \| \| 4 \| \| \| The Healthy Mummy Vanilla Flavoured Tummy Smoothie \| \| 5.0 \| 7 \| \| 4 \| \| \| The Healthy Mummy Vanilla Premium Blend Smoothie \| \| 5.0 \| 7 \| \| 4 \| \| \| The Kids Breakfast Shake Strawberry Flavour Shake \| \| 5.0 \| 7 \| \| 4 \| \| \| The Lady Shake Vanilla Meal Replacement Shake \| \| 5.0 \| 7 \| \| 4 \| \| \| The Lady Shake Vegan Formulated Meal Replacement Shake Sachets \| \| 5.0 \| 7 \| \| 4 \| \| \| The Man Shake Formulated Meal Replacement Shake Sachets \| \| 5.0 \| 7 \| \| 4 \| \| \| The Man Shake Vanilla Meal Replacement Shake \| \| 5.0 \| 7 \| \| 4 \| \| \| Tropeaka Natural Salted Caramel Flavour Lean Protein \| \| 5.0 \| 7 \| \| 4 \| \| \|  \|  \| \| \|  \| \|  \| \|  \| | | | | |  |  |  |
